# Can repeat injection provide clinical benefit in patients with cervical disc herniation and stenosis when the first epidural injection results only in partial response?

**DOI:** 10.1097/MD.0000000000004131

**Published:** 2016-07-22

**Authors:** Jung Hwan Lee, Sang-Ho Lee

**Affiliations:** a Department of Physical Medicine and Rehabilitation; b Department of Neurosurgery, Wooridul Spine Hospital, Seoul, Korea.

**Keywords:** cervical spine, herniated intervertebral disc, numeric rating scale, repeat injection, spinal stenosis, transforaminal epidural steroid injection

## Abstract

Epidural steroid injection (ESI) is known to be an effective treatment for neck or radicular pain due to herniated intervertebral disc (HIVD) and spinal stenosis (SS). Although repeat ESI has generally been indicated to provide more pain relief in partial responders after single ESI, there has been little evidence supporting the usefulness of this procedure. The purpose of this study, therefore, was to determine whether repeat ESI at a prescribed interval of 2 to 3 weeks after the first injection would provide greater clinical benefit in patients with partial pain reduction than intermittent ESI performed only when pain was aggravated. One hundred eighty-four patients who underwent transforaminal ESI (TFESI) for treatment of axial neck and radicular arm pain due to HIVD or SS and could be followed up for 1 year were enrolled. We divided the patients into 2 groups. Group A (N = 108) comprised partial responders (numeric rating scale (NRS) ≥ 3 after the first injection) who underwent repeat injection at a prescribed interval of 2 to 3 weeks after the first injection. Group B (N = 76) comprised partial responders who did not receive repeat injection at the prescribed interval, but received intermittent injections only for aggravation of pain. Various clinical data were assessed, including total number of injections during 1 year, NRS duration of <3 during 1 year (NRS < 3 duration), and time interval until pain was increased to require additional injections after repeat injection in Group A, or after first injection in Group B (time to reinjection). Groups A and B were compared in terms of total population, HIVD, and SS. In the whole population, HIVD subgroup, and SS subgroup, patients in Group A required significantly fewer injections to obtain satisfactory pain relief during the 1-year follow-up period. Group A showed a significantly longer time to reinjection and longer NRS < 3 than Group B did. Repeat TFESI conducted at 2- to 3-week intervals after the first injection in partial responders contributed to greater clinical benefit compared with intermittent TFESI performed only upon pain aggravation, with fewer TFESI sessions.

## 1 Introduction

Epidural steroid injection (ESI) has been known to be effective for the treatment of neck and radicular pain due to herniated intervertebral disc (HIVD) and spinal stenosis (SS).^[1–5]^ In patients who achieve only partial pain relief after the first injection, repeat ESI is indicated to provide better pain relief after reevaluation at 1- to 3-week intervals, while it is not usually recommended when there is no relief or complete relief.^[6,7]^

However, Arden et al^[8]^ revealed that repeat injection did not produce better clinical outcomes. In clinical practice, many physicians are concerned about the side effects related to repeated steroid administration and question whether repeat ESI improves clinical outcomes at long-term follow-up. Thus, physicians have been reluctant to perform repeat ESI, even in cases of partial response after the first injection. Thus, decisions about repeat ESI have frequently been made on the basis of physician's experience and preference rather than evidence supported by reports or standardized guidance.^[6,9]^ Therefore, it might be useful to provide information on whether or not repeat ESI at regular intervals would result in better clinical progression than intermittent injections performed only when pain became severe after partial clinical improvement at first injection.

Little literature exists regarding the clinical values of repeat ESI,^[6,10]^ and to our knowledge, there is no report on the clinical efficacy of repeat ESI in patients with cervical HIVD or SS. Previously, we demonstrated that repeat transforaminal epidural steroid injection (TFESI) performed at predetermined intervals obtained better clinical outcomes than TFESI randomly performed only upon pain aggravation in patients with lumbar HIVD or SS.^[11]^ However, we did not confirm that these results could be applied to patients with cervical HIVD and SS. TFESI has been reported as an effective treatment method in patients with axial neck and/or radicular pain due to medial and lateral disc herniation or stenosis, because this approach has an advantage to deliver the medication nearer the ventral epidural space or root sheath, which was the main source of pain.^[12–14]^ Thus, we performed this study to investigate whether repeat TFESI conducted at 2 to 3 weeks after the first injection in cases of partial pain reduction would lead to better clinical outcomes than those of intermittent TFESI performed only when pain was aggravated, in patients with cervical HIVD or SS.

## 2 Methods

### 2.1 Patient selection

This retrospective study was approved by the institutional review board of our hospital. Patients over 18 years of age who underwent their first TFESI for treatment of axial neck pain with radicular arm pain due to HIVD or SS between August 2014 and March 2015 and could be followed for 1 year were enrolled. These diagnoses were determined on the basis of clinical manifestation and radiological evaluation including magnetic resonance image (MRI). Patients who had clinically manifested neurological deficits, spinal cord compression seen on MRI, or history of cervical surgery were excluded. A radiologist who was an expert on spine pathology and blinded to the clinical characteristics of the patients in our study interpreted the MRI findings. HIVD was diagnosed if MRI showed localized displacement of intervertebral disc fragments beyond the intervertebral disc space. SS was diagnosed if MRI revealed canal or neural foraminal narrowing due to hypertrophy of ligaments or bony structures. Spinal cord compression could be detected by MRI, which showed narrowed or distorted normal cord structures or change in signal intensity inside the spinal cord. Two hundred thirty-two patients satisfied the enrollment criteria. Among them, 27 patients who had achieved satisfactory pain relief, to a score of <3 on the numeric rating scale (NRS), and 21 patients with no response (NRS reduction of <2 points) at the first injection were excluded. The 184 patients who showed partial response were included in this study. Partial response was defined as 3 points or more reduction in the NRS after first injection.

We divided the patients into 2 groups. Group A (N = 108) consisted of the patients who had a partial response to the first injection and underwent repeat ESI at 2 to 3 weeks. Group B (N = 76) consisted of the patients who had partial relief at the first injection but did not receive repeat injection even though NRS was not reduced below 3 at 2 to 3 weeks, and instead underwent intermittent injection only when pain was aggravated to the degree which treatment was required.

### 2.2 Data collection

Data such as age, gender, duration of pain, diagnosis (HIVD or SS), and location of the lesion were collected. Central or paracentral HIVD or SS was regarded as a medial lesion, and foraminal or extraforaminal HIVD or SS was regarded as a lateral lesion. NRS at pretreatment, number of injections during the 1-year follow-up period, NRS < 3 during the 1-year follow-up period (NRS < 3 duration), and time interval until pain was aggravated to require reinjection after repeat injection in Group A or after first injection in Group B (time to reinjection) were also assessed. Groups A and B were compared in terms of total population as well as each diagnosis subgroup (HIVD and SS).

### 2.3 Transforaminal ESI

All TFESIs were performed under computed tomography (CT) fluoroscopy by 1 physician (the first author, who was an expert in this procedure) using the same method published before.^[15]^ Injections were performed ipsilaterally, at a single level identified on MRI, which was compatible with the clinical manifestation. A patient was placed in the supine position, with his or her head turned slightly to the contralateral side from the injection site. Scout images were obtained through the desired cervical neural foramen, and the appropriate needle entry point was identified on the skin before skin preparation. The skin and subcutaneous tissue were anesthetized with 1 mL of 1% lidocaine at the entry point, and a 25-gauge spinal needle was partially inserted. The needle was adjusted and advanced toward the posterior aspect of the neural foramen using intermittent CT fluoroscopic guidance. The optimal placement of the needle tip was at the posterior aspect of the neural foramen.

During this procedure, multislice CT fluoroscopy images were utilized to guide the position of the needle. CT collimation was made with 4 mm × 3 mm slices that covered almost the entire intervertebral foramen height. This multislice CT fluoroscopy allowed us to locate the needle tip more exactly, not only at the X and Y axes, but also at the Z-axis. After confirming by CT images that the needle tip was placed in the desired location, 0.5 mL of contrast material was then slowly injected during a few seconds of continuous CT fluoroscopy. Images were obtained again after the end of the contrast injection to confirm that the injected contrast material was located inside the foramen. A mixture of 5 mg of dexamethasone and 1.0 cm^3^ of 0.5% lidocaine was prepared. After administration of a small volume (about 0.3 cm^3^) of the mixture and confirmation that there was no problem for few minutes afterwards, the rest of the mixture was slowly infused. After the needle was withdrawn, pressure was applied, and the patient was observed for an appropriate time before being released.

### 2.4 Statistical analysis

The SPSS Version 14.0 statistical package (SPSS, Inc., Chicago, IL) was used for statistical analysis. Because all data were normally distributed according to a Kolmogorov–Smirnov test, the parametric test was used. Chi-square test with Fisher exact test was used to compare gender ratio, proportion of diagnosis, number of injections during the 1-year follow-up period, and location of the lesion (medial lesion vs lateral lesion) between the 2 groups. Student *t* test was performed to determine differences in age, duration of pain, NRS at pretreatment, mean number of injections during 1 year, NRS < 3 duration, and time to reinjection. Results were considered statistically significant if the *P*-value was <0.05.

## 3 Results

### 3.1 Total population

Group A (N = 108) consisted of 55 men and 53 women, and Group B (N = 76) consisted of 41 men and 35 women. In Group A, 41 patients had HIVD and 60 had SS, and in Group B, 30 patients had HIVD and 56 had SS. No significant difference was found in terms of age, gender ratio, NRS at pretreatment, duration of pain, proportion of HIVD and SS, or location of lesions between the 2 groups.

In Group A, 2.32 ± 0.49 sessions were performed at 2- to 3-week intervals to obtain an NRS pain reduction of <3. The mean number of injections for Groups A and B were 2.53 ± 0.65 and 3.03 ± 1.02, respectively, during the 1-year follow-up period, which suggested that patients in Group A required significantly fewer injections during 1 year than Group B did, to accomplish satisfactory pain reduction. Group A also showed a significantly longer time to reinjection (5.56 ± 3.45 months) than Group B did (3.36 ± 2.05 months). The mean NRS of <3 duration during the 1 year was 9.65 ± 2.91 months in Group A and 6.20 ± 2.20 months in Group B, which meant that Group A had significantly longer duration of satisfactory pain remission than did Group B (Table 1).

**Table 1:**
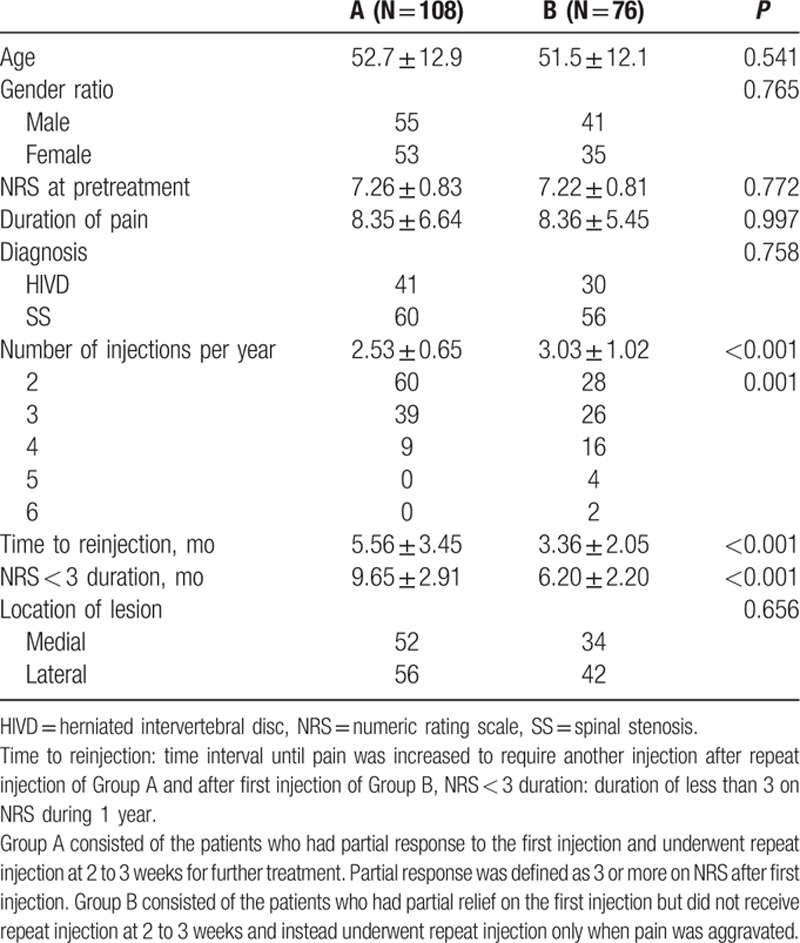
Comparison of clinical variables between Groups A and B.

A subgroup analysis was also performed in terms of gender, duration of pain (≤6 months vs >6 months), and lesion location (medial lesion vs lateral lesion) to identify which subgroup obtained clinical benefits from repeat injections. The same results were found, in that patients in Group A needed fewer injections during 1 year, and had longer time to reinjection and, and longer duration of NRS < 3 than Group B did, in all subgroups. This meant that clinical benefits were the results of repeat injection, not of other clinical and radiological variances.

No serious complications such as neurologic deficits, seizure, or loss of consciousness occurred. A few patients experienced minor side effects including transient dizziness, headache, or facial flushing, none of which required further treatment.

### 3.2 Herniated intervertebral disc

One hundred thirteen patients were diagnosed with HIVD, of whom 67 were in Group A and 46 were in Group B. No significant difference was found in terms of age, gender ratio, NRS at pretreatment, pain duration, and location of lesions between the 2 groups.

In Group A, 2.28 ± 0.45 sessions were performed at 2- to 3-week intervals to obtain an NRS reduction of <3. Group A and Group B received 2.48 ± 0.61 and 2.98 ± 0.95 injections on average during the 1-year follow-up period. Patients in Group A needed significantly fewer injections than those in Group B in order to accomplish an NRS of <3 during the 1-year follow-up period. The mean time to reinjection was 4.63 ± 2.90 months in Group A and 2.71 ± 1.25 months in Group B. Group A showed a significantly longer time to reinjection than Group B did. The mean NRS < 3 duration during 1 year was 9.93 ± 3.02 months and 5.72 ± 1.72 months in Groups A and B, respectively. Group A had significantly longer duration of satisfactory pain remission than Group B did (Table 2).

**Table 2:**
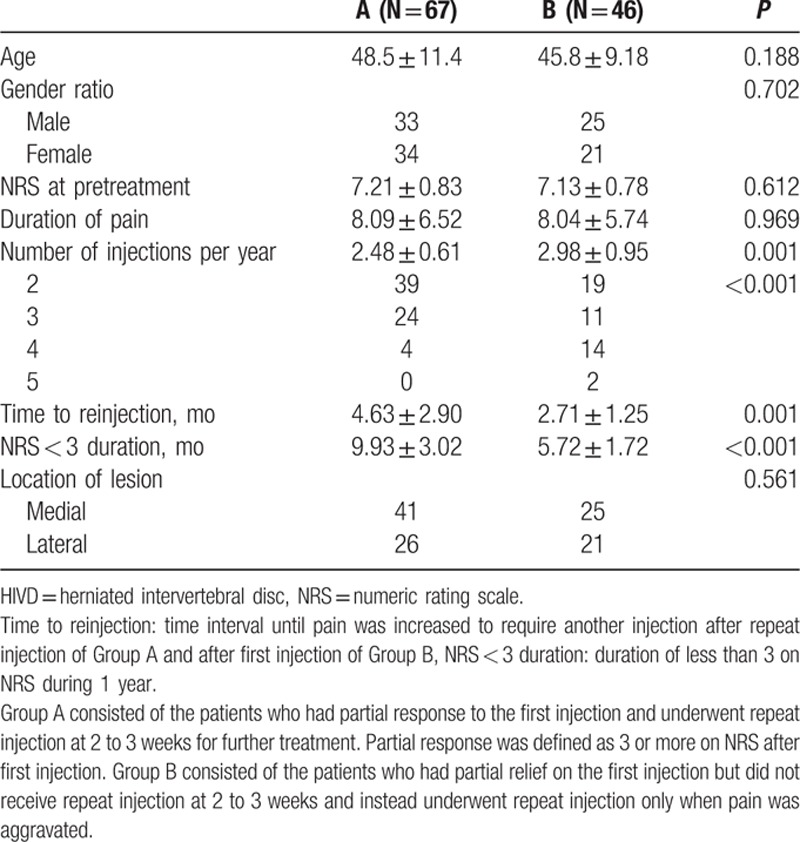
Comparison of clinical variables between Groups A and B diagnosed as HIVD.

### 3.3 Spinal stenosis

Seventy-one patients were diagnosed with SS, of whom 41 were in Group A and 30 in Group B. No significant difference was found in age, gender ratio, NRS at pretreatment, pain duration, or location of lesions between the 2 groups.

In Group A, 2.38 ± 0.54 sessions were performed at 2- to 3-week intervals to obtain a pain reduction NRS of <3. The mean number of injections during the 1-year follow-up period was 2.6 ± 0.70 and 3.10 ± 1.13 in Groups A and B, respectively. As a whole population and HIVD subgroup, patients in Group A in SS also required fewer injections than those in Group B to accomplish satisfactory pain relief during 1 year. The mean time to reinjection was 6.50 ± 3.80 months in Group A and 4.38 ± 2.61 months in Group B, which meant that Group A had a significantly longer time to reinjection than Group B did. The mean NRS < 3 duration during 1 year was 9.20 ± 2.70 months and 6.90 ± 2.67 months in Groups A and B, respectively. Group A had significantly longer duration of satisfactory pain remission than Group B did (Table 3).

**Table 3:**
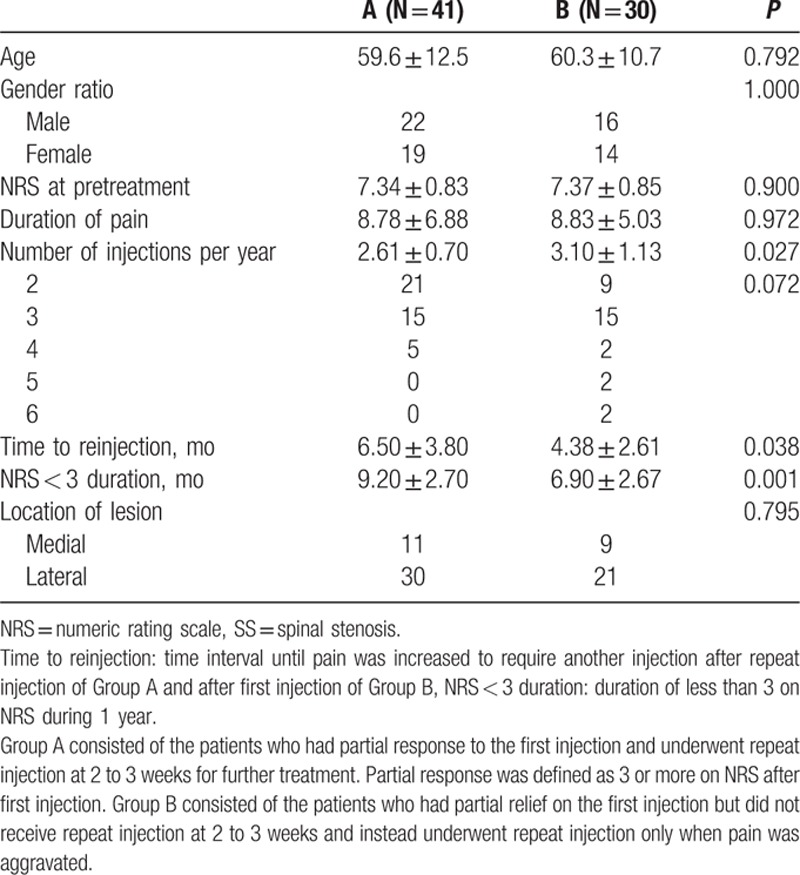
Comparison of clinical variables between Groups A and B diagnosed as SS.

## 4 Discussion

Repeat ESI has been recommended and, therefore many physicians have conducted this procedure to provide more pain relief in partial responders after single ESI.^[6,7,16]^ However, there has been little evidence as to whether repeat injection actually provides greater and more prolonged pain relief. Even the literature regarding repeat injection has not distinctly demonstrated as to when repeat injection was required or whether repeat injection at established intervals has better clinical effects than intermittent injection performed at random intervals.^[8,10,17–19]^ On the other hand, 1 study reported that repeat injection at 3 to 6 weeks did not have prolonged or cumulative effects.^[8]^

Because there is insufficient evidence in favor of repeat injection, as well as concerns about the potential side effects related to repeated steroid administrations, many physicians were opposed to repeat injection or were not sure whether repeat injection provided patients with more clinical benefits over disadvantages in case of partial response after first injection. Recently, we demonstrated that repeat TFESI at predetermined intervals was useful to obtain more prolonged pain reduction, compared with randomly performed TFESI in patients with lumbar HIVD and SS.^[11]^ Although this article provided evidence to support the clinical usefulness of repeat injection, it was not certain that this result could be applied to cervical lesions. This motivated us to begin a similar study in patients with cervical diseases. Due to the serious complications related to cervical TFESI, however, many physicians are reluctant to perform repeat TFESI in patients with cervical lesions.

In this study, the mean number of injections during the 1-year follow-up period was smaller in the repeat injection group (Group A) than in the intermittent injection group (Group B). The NRS < 3 duration and time to reinjection for control of aggravated pain were also significantly longer in Group A than in Group B. In addition, Group A obtained effective pain reduction of NRS < 3 with fewer than 3 sessions (2.32 ± 0.49). During the 1-year follow-up period, only 9 (8.3%) of 108 patients in Group A underwent more than 3 treatment sessions, while 22 (28.9%) of 76 patients in Group B required more than 3 treatment sessions. These results suggested that if the first injection provided only partial pain relief, repeat injection at 2 to 3 weeks reduced pain over a longer time period than intermittent, random injection, as well as decrease the possibility of side effects related to steroid accumulation by reducing treatment sessions. This benefit of repeat injection was found in each disease subgroup, HIVD and SS, irrespective of disease location and also found irrespective of other clinical and radiological factors. These longer effects might be the result of cumulative clinical benefits and restoration of benefits that could subsequently be diminished after the first injection.^[10,11]^

We needed to choose the same technique (CT fluoroscopy-guided TFESI) in all subjects to avoid the influences that might come from a different approach method or inaccurate drug administration by a blinded method. We tried to remove other factors such as different approach methods (interlaminar vs transforaminal) or different radiological guidance (C-arm fluoroscopy vs CT fluoroscopy) that could affect clinical results except repeat injection at regular intervals as much as possible, because increased clinical efficacy could be interpreted as a cumulative effect obtained by repeat injection rather than an inappropriate or different treatment method.

Notably, cervical TFESI could be associated with more serious side effects such as neurologic deficits, because particulate corticosteroid delivered by the transforaminal approach could be inadvertently injected into vascular structures and produce aggregated embolus, which could lead to subsequent cerebellar, brainstem, or spinal cord infarct.^[20,21]^ Intraarterial needle penetration could frequently occur, even when the needle was placed in an appropriate position, because vascular structures, including the radicular and vertebral arteries were closely located in the needle advancement route.^[20,22]^ Intravasation of local anesthetics could also cause seizure or loss of consciousness.^[22–25]^ No serious side effects were observed in our study. This might be explained by 2 hypotheses. First, we used a dexamethasone, nonparticulate steroid. This did not produce an aggregated embolus that could lead to serious side effects, including neurologic complications. Second, we used CT fluoroscopy guidance, which could provide superior anatomical resolution in the transverse plane, allowing precise placement of the needle tip with a minimal readjustment. Consequently, it helped us avoid penetrating important vascular structures such as the jugular, vertebral, carotid, or radicular vessels, while advancing the needle into the posterior neural foramen.^[15,26,27]^ Therefore, we suggested that physicians did not need to avoid repeat cervical TFESI for the concerns of safety if a nonparticulate steroid was administered with an appropriate needle approach. On the other hand, repeat injection produced cumulative and prolonged clinical effects, which further helped us avoid unnecessary extensive surgical treatment, usually considered the next treatment strategy after failure of injections.

The present study had limitations related to its retrospective design. First, only patients who could be followed up for 1 year were chosen, and those with partial response at the first injection who were lost to follow up before 1 year were not included or analyzed in this study. Second, some patients were not included because they underwent surgery before completion of the 1-year follow-up period due to aggravated pain. Third, we used only 1 pain score, the NRS, as a clinical evaluation method, and did not measure functional score or patients’ satisfaction score. This was overly simplistic and did not take into account various aspects of clinical outcomes. We suppose that a prospective cohort study using more clinical assessment methods would provide more informative and supportive evidence for repeat ESI at regular intervals.

## 5 Conclusion

Repeat TFESI conducted at 2 to 3 weeks after the first injection in cases of partial pain reduction contributed to prolonged clinical benefits with fewer steroid administrations than intermittent TFESI randomly conducted in patients with cervical HIVD or SS.
